# A Single Intramuscular Vaccination of Mice with the HSV-1 VC2 Virus with Mutations in the Glycoprotein K and the Membrane Protein UL20 Confers Full Protection against Lethal Intravaginal Challenge with Virulent HSV-1 and HSV-2 Strains

**DOI:** 10.1371/journal.pone.0109890

**Published:** 2014-10-28

**Authors:** Brent A. Stanfield, Jacque Stahl, Vladimir N. Chouljenko, Ramesh Subramanian, Anu-Susan Charles, Ahmad A. Saied, Jason D. Walker, Konstantin G. Kousoulas

**Affiliations:** Division of Biotechnology & Molecular Medicine and Department of Pathobiological Sciences, School of Veterinary Medicine, Louisiana State University, Baton Rouge, Louisiana, United States of America; Cincinnati Childrens Hospital Medical Center, United States of America

## Abstract

Herpes Simplex Virus type-1 (HSV-1) and type-2 (HSV-2) establish life-long infections and cause significant orofacial and genital infections in humans. HSV-1 is the leading cause of infectious blindness in the western world. Currently, there are no available vaccines to protect against herpes simplex infections. Recently, we showed that a single intramuscular immunization with an HSV-1(F) mutant virus lacking expression of the viral glycoprotein K (gK), which prevents the virus from entering into distal axons of ganglionic neurons, conferred significant protection against either virulent HSV-1(McKrae) or HSV-2(G) intravaginal challenge in mice. Specifically, 90% of the mice were protected against HSV-1(McKrae) challenge, while 70% of the mice were protected against HSV-2(G) challenge. We constructed the recombinant virus VC2 that contains specific mutations in gK and the membrane protein UL20 preventing virus entry into axonal compartments of neurons, while allowing efficient replication in cell culture, unlike the gK-null virus, which has a major defect in virus replication and spread. Intramuscular injection of mice with 10^7^ VC2 plaque forming units did not cause any significant clinical disease in mice. A single intramuscular immunization with the VC2 virus protected 100% of mice against lethal intravaginal challenge with either HSV-1(McKrae) or HSV-2(G) viruses. Importantly, vaccination with VC2 produced robust cross protective humoral and cellular immunity that fully protected vaccinated mice against lethal disease. Quantitative PCR did not detect any viral DNA in ganglionic tissues of vaccinated mice, while unvaccinated mice contained high levels of viral DNA. The VC2 virus may serve as an efficient vaccine against both HSV-1 and HSV-2 infections, as well as a safe vector for the production of vaccines against other viral and bacterial pathogens.

## Introduction

Genital herpes has a very high global prevalence and disease burden. Recent seroprevalence studies for the years 2005–2010 indicate that 1 out of 2 adults in the United States ages 14–49 years old are latently infected with herpes simplex type-1 (HSV-1) [1]. Most infected individuals experience frequent, but asymptomatic episodes of virus shedding that contribute to high virus transmission rates [2]–[4]. An increasing number of HSV-1 rather than HSV-2 infections are being observed in clinical cases involving genital infections [5]. Importantly, genital HSV infection is considered a risk factor for acquiring human immunodeficiency virus infection (HIV) [6]–[11], and in some geographical areas HSV-2 infection may be a contributing factor to 30–50% of new HIV infections [12], [13]. A successful vaccination strategy against HSV-2 infection is predicted to have a dramatic global impact on HIV spread, prevention of genital clinical disease and neonatal infections [14]–[16]. Prior HSV immunity may confer only partial protection against HSV re-infection and the appearance of clinical disease symptoms [2], [17]. Adaptive immune responses, particularly tissue specific CD4^+^ and CD8^+^ T cells are crucial for controlling HSV infections and clearing the virus after initial infection. These T cell responses are also important in containing the virus in a latent state in ganglionic or dorsal neurons, as well as for controlling the virus after reactivation from latency [18]–[24]. Humoral responses have also been implicated in playing an important role in controlling HSV infectivity, spread, and the rate of reactivation from latency [25]–[27].

A number of vaccine approaches and candidates have been evaluated in laboratory animals and humans including purified peptides, recombinant glycoprotein subunits, inactivated, live attenuated, replication competent and replication defective whole virus, as well as DNA-based vaccines administered via different routes of immunization (reviewed in: [28]–[32] In a double-blind controlled, randomized efficacy field trial of a HSV-2 glycoprotein D (gD-2) subunit vaccine adjuvanted with A04 (Herpevac Trial) in 8323 women, it was found that the vaccine was 82% protective against HSV-1 genital disease, but offered no significant protection against HSV-2 genital disease [33]. This protection correlated with induction of neutralizing antibody against gD-2, while cellular immune responses did not appear to be involved in the observed protection [34], [35]. A newer subunit vaccine approach currently in phase I/IIa clinical trials is based on an attempt to generate a balanced T cell and antibody response through the use of T-cell epitopes derived from the ICP4 protein and antibody generated by the gD-2 glycoprotein in conjunction with the proprietary adjuvant Matrix-M [30].

In principle, live attenuated vaccines have distinct advantages over subunit and inactivated vaccines, primarily because replication of the pathogen allows for the entire repertoire of pathogen-specific antigen expression. Given the 83% nucleotide identity shared by both HSV-1 and HSV-2 genomes [36], cross protective immunity may be achieved by a single safe and efficacious vaccine expressing a large enough repertoire of cross-protective antigens. Attempts at generating a live attenuated HSV vaccine have focused on the preparation of attenuated viruses that can generate robust immune responses, while minimizing potential virulence in the host. Generally, entire genes that play important roles in the virus lifecycle have been deleted or otherwise modified to attenuate the virus and allow a more robust production of humoral and cellular immune responses. Viral genome modifications include deletions in glycoprotein E (gE) [37], [38], multiple deletions in γ34.5, UL55-56, UL43.5, US10-12 [39], UL5, UL29, UL42, ICP27 genes [40]–[43], deletion of ICP0- [44] and the UL9 gene [45]–[48]. Other live virus vaccines under study include the HSV-1 virus CJ9-gD engineered to overexpress gD1 and having a dominant negative mutation to prevent virus replication. This vaccine strain has been reported to protect guinea pigs from HSV-2 intravaginal challenge, with marked reduction in vital titer and lesion formation [47].

Generation of a safe and effective replication competent HSV-1 virus is important to not only vaccinate against acquiring HSV infection and reduce HIV prevalence, but also a safe vaccine vector could be utilized for expression of heterologous antigens from other pathogens. HSV has many non-essential genes and can stably carry large fragments of foreign DNA. This genetic flexibility is ideal for the expression of antigens specific to other pathogens [49], [50]. Recently, a recombinant HSV expressing granulocyte monocyte colony stimulating factor (GM-CSF), a potent chemokine functioning in the maturation of macrophages, has been used in combination with other chemotherapeutics for the treatment of squamous cell cancer of the head and neck with promising phase I/II results [51]. FDA approval for this particular HSV vaccine therapy for melanoma is expected to pave the way for the use of live-attenuated HSV-based vectors for vaccination against HSV and other pathogens.

Previously, we have shown that a gK-null virus was unable to infect ganglionic neurons and establish latency after ocular infection of mice [52], [53]. We capitalized on the attenuated properties of the gK-null virus and showed that intramuscular vaccination of mice with the gK-null virus conferred significant cellular immune responses and protection against intravaginal challenge of mice with either virulent HSV-1(McKrae) or HSV-2(G) viruses [54]. To further improve on this vaccination approach, we constructed the VC2 mutant virus with specific deletions within the genes coding for glycoprotein K (gK) and UL20. The VC2 virus contains the gKΔ31-68 mutation that prevents the virus from infecting ganglionic neurons after ocular infection in mice [55]. In contrast to the gK-null virus that requires replication in the complementing cell line VK302 that expresses gK [54], the VC2 virus can replicate efficiently in infected Vero cells achieving titers similar to that of the wild-type HSV-1(F) parental virus in cell culture. Herein, we report that a single intramuscular vaccination with the VC2 virus was very well tolerated at a high infectious dose (10^7^ PFU), produced a robust humoral and cell-mediated immune response and conferred 100% protection against lethal intravaginal challenge with either HSV-1 (McKrae) or HSV-2 (G) viruses.

## Results

### Construction and characterization of the VC2 vaccine virus

**Figure 1:**
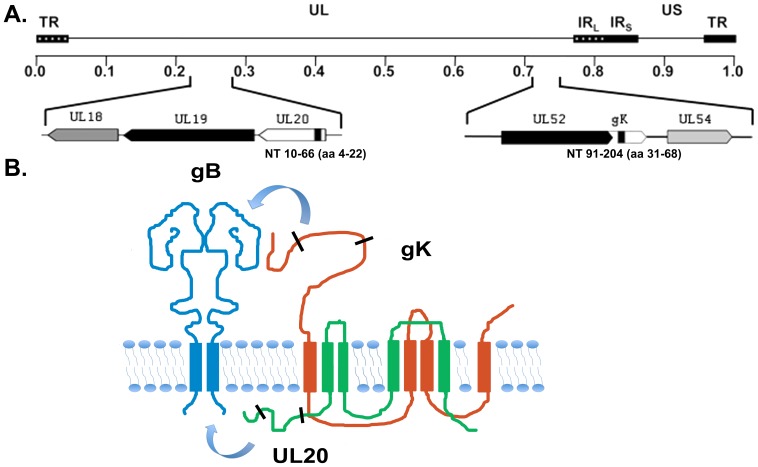
Schematic of the construction of VC2. (A) The top line represents the prototypic arrangement of the HSV-1 genome, with the unique long (UL) and unique short (US) regions flanked by the terminal repeat (TR) and internal repeat (IR) regions. Shown below are the expanded genomic regions which encompass the open reading frames of UL20 and glycoprotein K. In black are the approximate deletions within their respective genes. (B) A graphical depiction of the gK UL20 complex interacting with gB. Areas between the black lines on the graphical depiction represent the aproximate location of the deletion in their respective genes.

The VC2 recombinant virus was constructed utilizing the two-step double-Red recombination protocol implemented on the cloned HSV-1(F) genome [55] in a bacterial artificial chromosome (BAC) plasmid [56], as we have described previously [57], [58], and detailed in the Materials and Methods section. The VC2 virus contains the gKΔ31-68 deletion (37 aa; gK aa 31–68) in the amino terminus of gK that prevents the virus from entering into ganglionic neurons after infection via the ocular route [55], as well as a deletion of the amino-terminal 19 amino acids of the UL20 virus (Fig. 1A). Next generation whole genome sequencing was performed to validate that only intended mutations were induced into the HSV-1(F) BAC. A total sequence output of Q20 quality that is derived from the predicted per-base quality scores and corresponds to an error rate of 1% generated 266× and 443× coverage for the two biological replica samples sequenced. A total of 37 nt changes and 13 of that caused aa differences were detected in comparison to the Gene bank submission GU 734771 of human herpes virus type 1 (strain F, complete genome), as we have reported previously for other HSV-1(F) BAC mutant viruses [59]. Overall, there were no nucleotide changes between the parental HSV-1(F) BAC and the derived VC2 mutant virus with the exception of the engineered deletions within the UL20 and gK genes.

**Figure 2:**
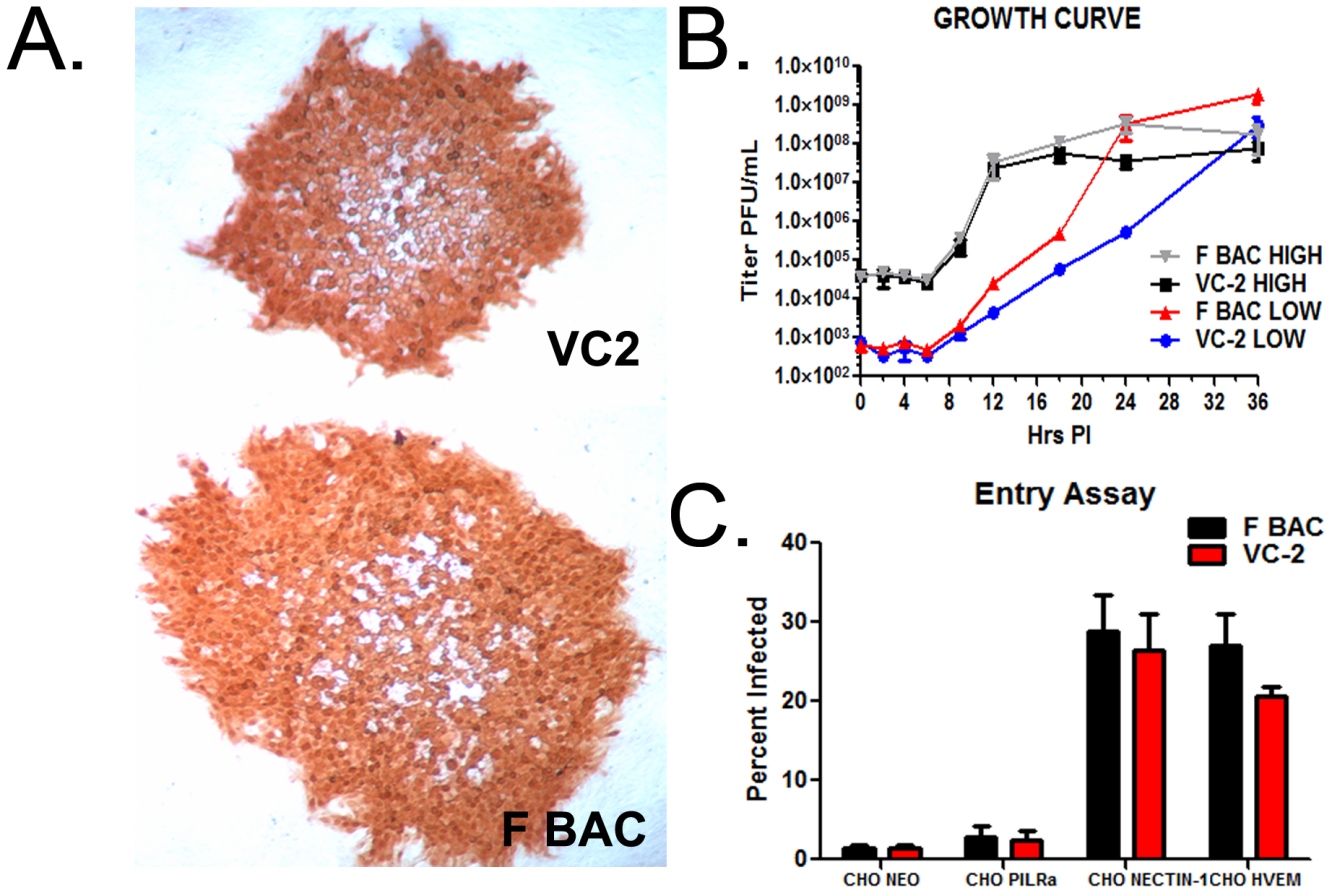
In-vitro analysis of the replication and entry characteristics of VC2 vs F BAC. (A) Plaque morphology of VC2 vs F BAC on Vero cells 48 hours post infection visualized by IHC and developed with NovaRed substrate. (B) Growth curve representative of the replication kinetics of VC2 vs F BAC at both and low (0.1) and high (5) MOI. Samples collected at times 0, 2, 4, 6, 9, 12, 18, 24, and 36 hours post infection titrated on Vero cells. (C) Entry assay depicting VC-2 vs F BAC into Chinese hamster ovary (CHO) cells expressing known herpes virus entry receptors PILR-α, nectin −1, HVEM, and NEO for a negative control.

We have previously shown that the amino termini of both gK and UL20 interact with gB and that these interactions modulate virus-induced cell fusion mediated by the gK/UL20 protein complex [57], [60], [61] (Fig. 1B). The UL20Δ4-22 mutation does not affect virus replication, although it produces a syncytial phenotype (not shown). However, the simultaneous presence of the gKΔ31-68 and UL20Δ4-22 deletions produce non-syncytial plaques, which were 30–40% smaller in size than those of the parental virus (Fig. 2A). The VC2 virus replicated as efficiently as the parental wild-type HSV-1(F) BAC virus at a MOI of 5. At low MOI (0.1), VC2 replicated with slower kinetics, but achieved similar peak virus titers by 36 hpi (Fig. 2B). The VC2 and HSV-1(F) BAC viruses exhibited similar entry efficiencies into Chinese hamster ovary (CHO) cells expressing the HSV-1 receptors Nectin-1, and HVEM (Fig. 2C). Both HSV-1(F) and VC2 failed to enter into paired immunoglobulin receptor-α (PILRα), expressing CHO cells, as we have reported previously for HSV-1(F) in comparison to HSV-1(McKrae) [62].

### Vaccination and intravaginal challenge of VC2 vaccinated mice with HSV-1(McKrae) and HSV-2(G) viruses

**Figure 3:**
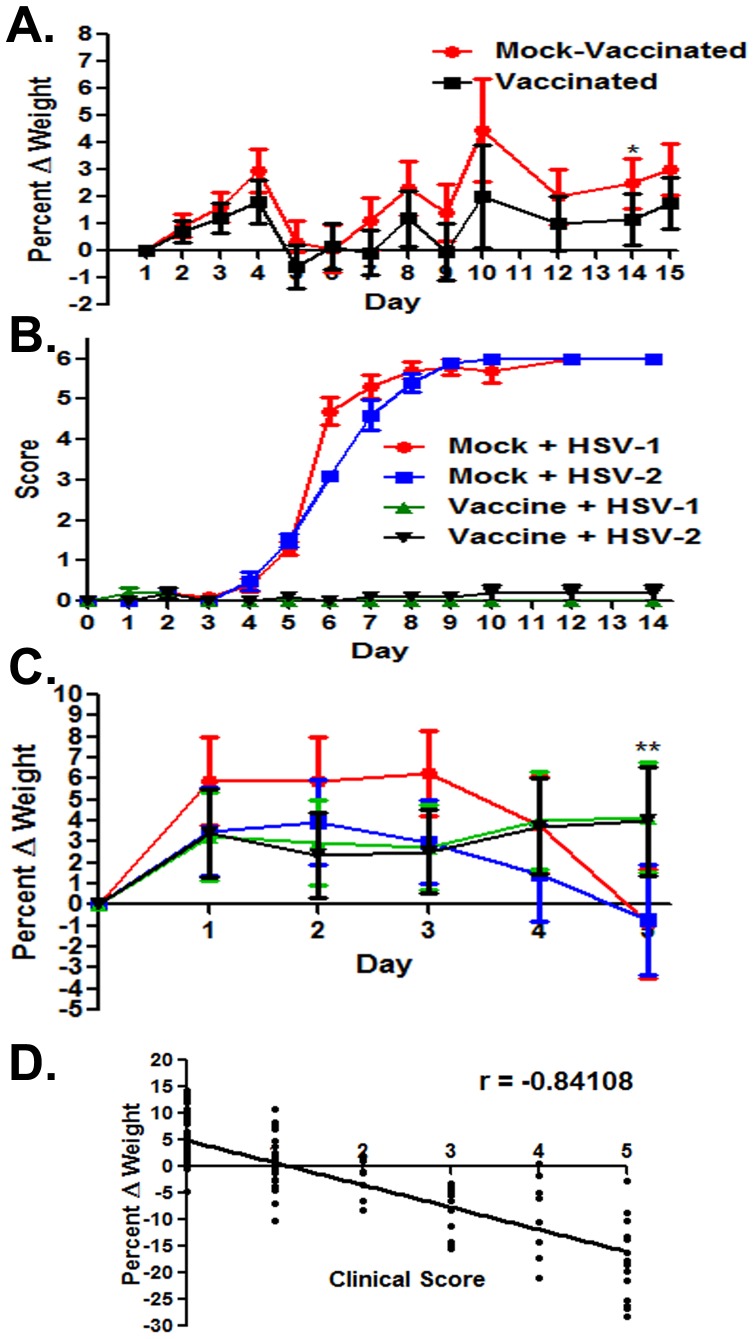
Pre and post challenge morbidity. (A) Pre challenge percent change in weight in mock vs vaccinated animals. Percentages normalized to the initial weight at day 0. ** p≤0.01. Bars represent the 95% confidence interval about the mean. Statistical comparison conducted by SAS using Proc Mixed Type 3 Tests of Fixed Effects. (B) Clinical scoring of mice previously receiving mock IM vaccination or 10^7^ PFU IM vaccination of VC2. Two groups (Mock and Vaccinated n = 10 each) received a intravaginal challenge of 10^6^ PFU of HSV-2 (G) and 2 groups (Mock and Vaccinated n = 10 each) received a intravaginal challenge of 10^6^ PFU of HSV-1 (McKrae). Mice were scored on a scale of 0–6 (0 = no disease, 1 = ruffled fur and generalized morbidity, 2 = mild genital erythema and edema, 3 = moderate genital inflammation, 4 = genital inflammation with purulent discharge, 5 = hind limb paralysis, 6 = death). (C) Percent change in weights post challenge in Mock vs vaccinated animals challenged with either HSV-1 (McKrae) or HSV-2 (G). Percentages normalized to the initial weight at day 0. ** p≤0.01. Bars represent the 95% confidence interval about the mean. Statistical comparison conducted by SAS using Proc Mixed Type 3 Tests of Fixed Effects. (D) Correlation between percent change in weight VS. Clinical score of unvaccinated mice Gaussian Approximation p<0.0001 Spearman r = −0.84108.

**Figure 4:**
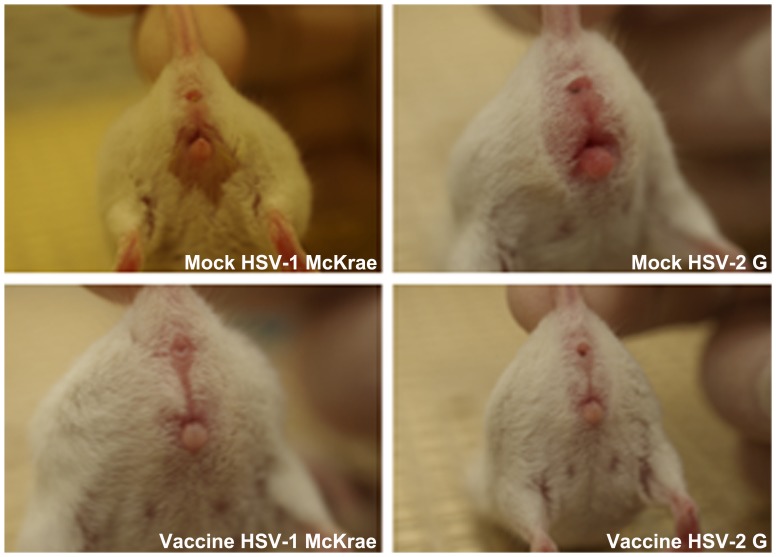
Pathogenesis post challenge. Disease pathology among mock (top two panels) and vaccinated (bottom two panels) animals challenged with either HSV-1 (McKrae) or HSV-2 (G) 5 days post challenge. HSV-1(McKrae) and HSV-2(G) infected mice exhibited similar disease progression and pathology in the mock groups (top two panels). Vaccinated mice (bottom two panels) did not exhibit any clinical disease over the observation period post challenge. Mild disease symptoms included ruffled fur, hunching posture, inflammation and redness of vagina (top right). More serious manifestations included purulent vaginal discharge (top left).

Initial safety experiments indicated that the VC2 virus did not produce significant clinical disease symptoms after intranasal or intramuscular injection of 10^7^ PFU per mouse, and there was no viral DNA detected by PCR in either dorsal or trigeminal ganglia from these mice (not shown). The vaccine strategy involved intramuscular injection of 10^7^ PFU of the VC2 virus in each mouse followed by treatment of mice at day 15 post vaccination via intramuscular injection of Depo Provera, as described previously [54], and intravaginal challenge with 10^6^ PFU of either HSV-1(McKrae) or HSV-2(G) viral strains at day 21 post vaccination. VC2 vaccinated mice were monitored for clinical disease symptoms and the body weights were also monitored daily. No significant clinical disease symptoms were noted throughout the 15 day observation period. Also, no significant differences in body weights of vaccinated versus mock-vaccinated animals were observed except on day 14 post vaccination (p<0.05) (Fig. 3A). Following vaginal challenge with either HSV-1(McKrae) or HSV-2(G), infected mice were observed daily for disease manifestations. Clinical scores and weight measurement were recorded for all live mice for fifteen days following challenge,. Mock-vaccinated animals showed pronounced, time-dependent increase in clinical disease symptoms and a significant concomitant decrease in weight by day 5 post challenge (Fig. 3: B, C). Analysis of clinical scores with the corresponding changes in weights of unvaccinated mice revealed a strong correlation between increasing clinical scores and decreasing body weights (Fig. 3D). This correlation analysis revealed that the observed difference in vaccinated versus mock-vaccinated animals on day 14 post vaccination was not indicative of significant overall morbidity. Disease symptoms in the mildest cases consisted primarily of hair loss, hunched posture and fur ruffling (not shown). More advanced disease symptoms included significant vaginal and peri-anal erythema and edema and purulent discharge (Fig. 4). As noted previously [54], HSV-1(McKrae) caused significant clinical disease approaching that observed in HSV-2(G) infections (Fig. 4).

**Figure 5:**
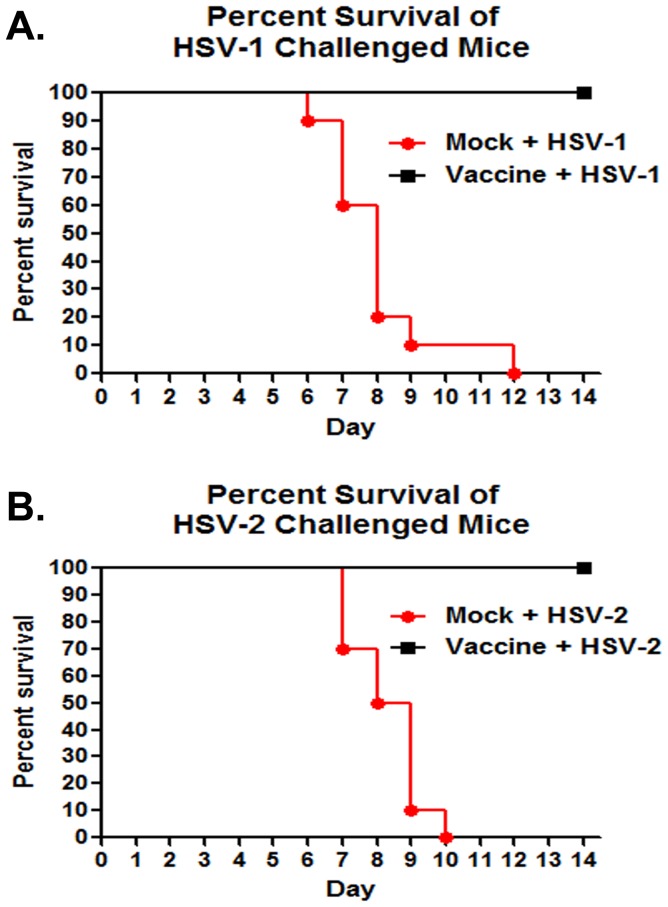
Kaplan-Meier survival curves. Vaccinated and mock-vaccinated mice in challenge groups were challenged thorough the intra-vaginal route with 10^6^ PFU of HSV-1 McKrae (A) or HSV-2G (B) 21 days post primary vaccination and observed for 14 days. One hundred percent of the vaccinated animals in the HSV-1 and HSV-2 challenged group survived, while 100% of the mock-vaccinated animals died. (A) A statistically significant difference was observed between the vaccinated and mock-vaccinated groups (p<0.0001) using the Gehan-Breslow-Wilcoxin test. (B) A statistically significant difference was observed between the vaccinated and mock-vaccinated groups (p<0.0001) using the Gehan-Breslow-Wilcoxin test.

**Figure 6:**
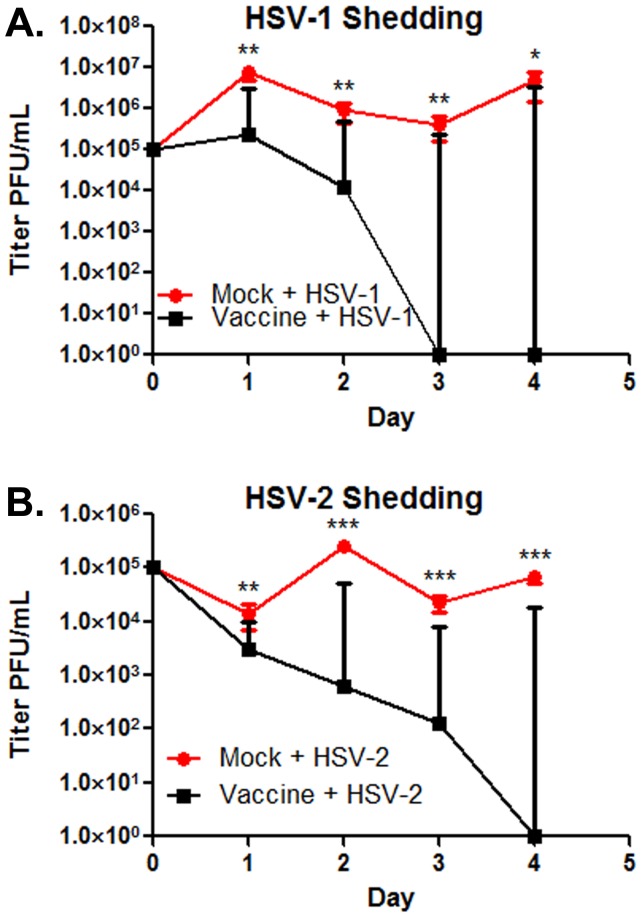
Vaginal shedding post challenge. (A) HSV-1 shedding post challenge in mock vs vaccinated animals. (B) HSV-2 shedding post challenge in mock vs vaccinated animals. Significant differences in shed titers noted as * p≤0.05, ** p≤0.01, or *** p≤0.0001. Bars represent the 95% confidence interval about the mean. Statistical comparison conducted by SAS using The Mixed Procedure Type 3 Tests of Fixed Effects.

Vaccinated mice were completely protected against lethal challenge. Mice in the mock-vaccinated group started dying on day 6 for the HSV-1 challenged group, and day 7 in the HSV-2 challenged group. In both groups of mice, challenged with either HSV-1 or HSV-2, protection against lethal challenge was significantly higher in vaccinated than mock-vaccinated animals (p<0.0001) (Fig. 5). Vaginal shedding was assessed for 4 days following challenge. Significant reductions in virus shedding were observed on all days post challenge in the HSV-1 (days 1, 2: p = 0.0002; day 3: p = 0.008; day 4: p = 0.0244): and HSV-2 group of mice (day 1: p = 0.0014; days, 3–4: p<0.0001). In both cases, vaccinated animals did not shed any virus after day 4 post challenge. Overall, lower viral titers were recovered from the vaginas of the HSV-2 than the HSV-1 challenged mice (Fig. 6: A, B).

To determine whether HSV-1 (McKrae), or HSV-2(G) were able to infect ganglionic or dorsal root neurons and establish latent infection, challenged mice were immunocompromised by injection of cyclophosphamide followed by injection of dexamethasone at 100 days post challenge. No infectious virus was recovered from vaginal swabs of vaccinated mice treated with cyclophosphamide and dexamethasone which is known to chemically induce reactivation of virus from latency [63]. Similarly, there was no viral DNA detected for either HSV-1(McKrae) or HSV-2(G) in the extracted neuronal tissues by virus type-specific quantitative PCR (qPCR) for the vaccinated mice at the lowest possible detection limit of approximately 3 viral genomes (see Materials and Methods section). In contrast, tissues from mock-vaccinated animals revealed the presence of high copy numbers of either HSV-1(McKrae) or HSV-2(G) viral DNA in the respective animal groups (Table 1).

**Table 1:** qPCR of dorsal root ganglia from vaccinated animals.

HSV-1 Challenge	HSV-1 gD	HSV-2 gD	HSV-2 Challenge	HSV-1 gD	HSV-2 gD
Positive Control	>1.0 × 10^6^	ND	Positive Control	ND	>1.0 × 10^6^
161	ND	ND	171	ND	ND
162	ND	ND	172	ND	ND
163	ND	ND	173	ND	ND
164	ND	ND	174	ND	ND
165	ND	ND	175	ND	ND
166	ND	ND	176	ND	ND
167	ND	ND	177	ND	ND
168	ND	ND	178	ND	ND
169	ND	ND	179	ND	ND
170	ND	ND	180	ND	ND

qPCR results from sacral dorsal root ganglia excised from vaccinated mice challenged with either HSV-1 (McKrae) or HSV-2 (G). Positive controls were sacral dorsal root ganglia excised from unvaccinated mice challenged with either HSV-1 (McKrae) or HSV-2 (G). Samples below the limit of detection are designated as ND. The qPCR assays specific for either HSV-1 and HSV-2 viral DNA detected as low as 3 viral DNA copies/µL (see Materials and Methods).

### VC2-induced humoral and cellular immune responses

**Figure 7:**
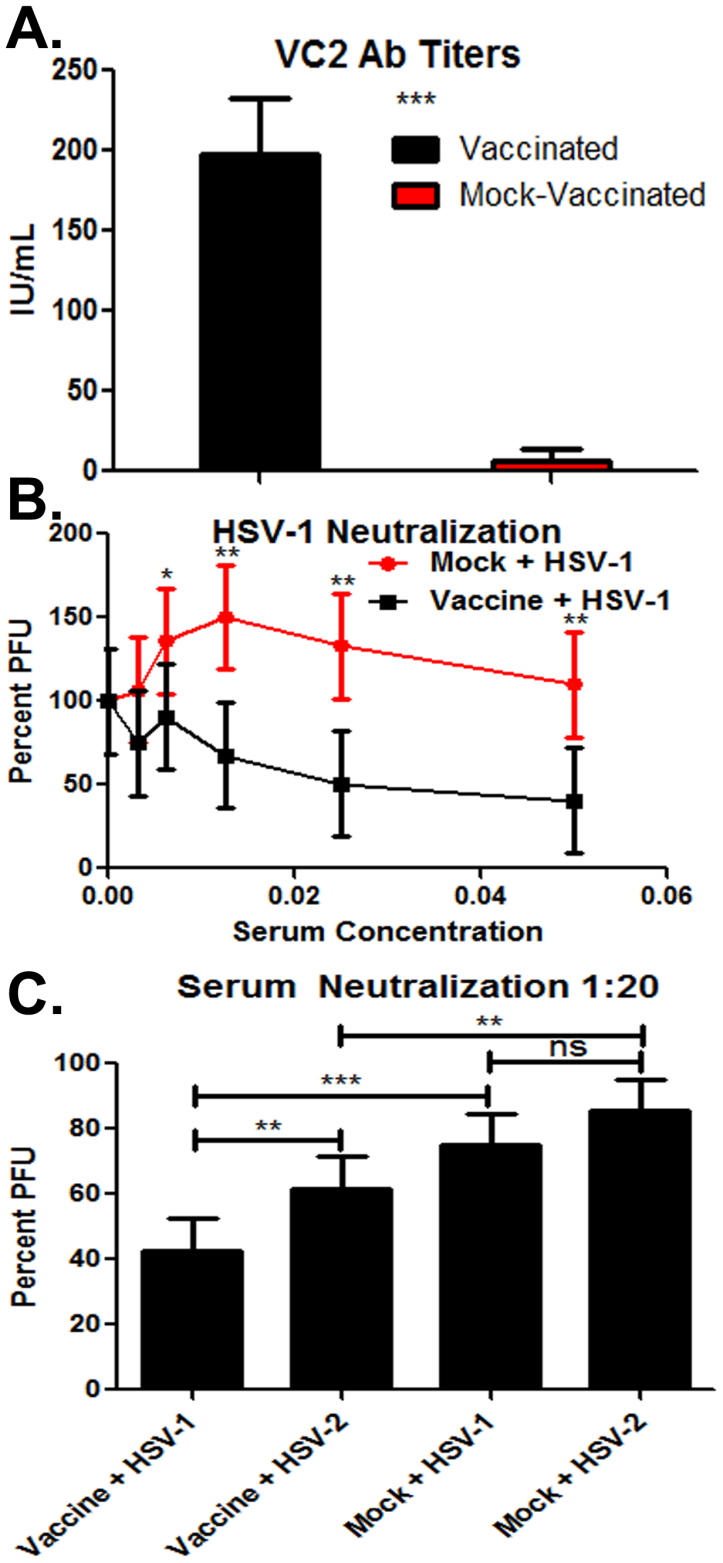
In-vitro analysis of humoral immune response. (A) Colorimetric ELISA based analysis of HSV-1 reactive polyclonal IgG produced 21 days post vaccination n = 20. Statistical comparison conducted by SAS using the T test Procedure. Bars represent the 95% confidence interval about the mean (B) Titration of serum neutralizing fixed PFU of HSV-1 (McKrae) normalized to a no serum control n = 5. Significant reduction in PFU observed 1∶160, 1∶80, 1∶40, and 1∶20 dilutions of the sera. Statistical comparison conducted by SAS using The Mixed Procedure and Differences in Least Squares Means. Bars represent the 95% confidence interval about the mean. (C) Cross reactive neutralization of HSV-1 (McKrae) and HSV-2 (G) at a 1∶20 dilution of sera from vaccinated and mock inoculated mice. Percent neutralization normalized to no serum controls. Statistical comparison conducted by SAS using The Mixed Procedure and Differences in Least Squares Means. Bars represent the 95% confidence interval about the mean. Significant differences noted as * p≤0.05, ** p≤0.01, or *** p≤0.0001.

To assess the relative levels of HSV-1 specific antibodies raised by the VC2 vaccination a commercially available ELISA was utilized to measure the relative quantity of HSV-specific IgG. In this ELISA, plates were coated with HSV-1-infected cell extracts and HSV-1 bound antibodies were quantified by colorimetry (see Materials and Methods). All VC2 vaccinated mice produced HSV-1-specific antibodies, while none of the mock-vaccinated animals produced detectable anti-HSV antibodies (Fig. 7A). The VC-2-induced antibodies were tested for ability to neutralize HSV-1(McKrae) virus. Antisera from five vaccinated and mock-vaccinated mice were individually tested at serial dilutions for ability to neutralize the virus, as described in Materials and Methods. Substantial neutralization of HSV-1(McKrae) was noted at 1∶160, 1∶80, 1∶40 and 1∶20 dilutions of each mouse serum (Fig. 7B). To further investigate the neutralization activities of these sera, the 1∶20 dilution was chosen to test for ability to neutralize both HSV-1(McKrae) and HSV-2(G). Significant differences were observed between the neutralization of HSV-1(McKrae) and HSV-2(G) by sera of VC2-vaccinated animals in comparison to the mock-vaccinated mice. However, no significant differences were observed between the mock-vaccinated groups (Fig. 7C).

**Figure 8:**
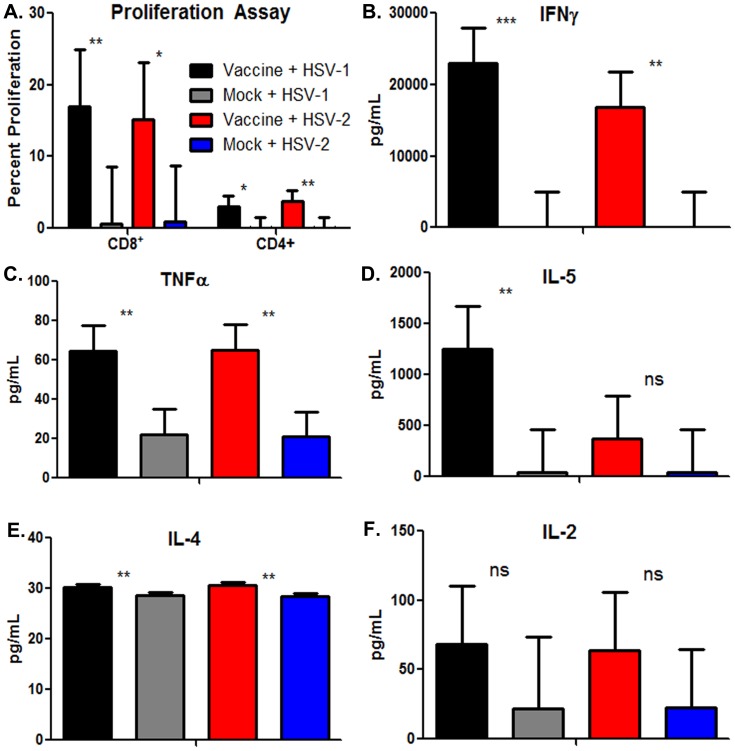
In-vitro analysis of cellular immune response. (A) Proliferation of CD4^+^ vs CD8^+^ T cells from mice which received the vaccine and mice which were mock injected and stimulated with pooled HSV-1 or HSV-2 peptides. (B–F) CBA analysis of secreted cytokine concentration in cell culture supernatant from T cell proliferation assay. Statistical comparison conducted by SAS using The Mixed Procedure and Differences in Least Squares Means. Bars represent the 95% confidence interval about the mean. Significant differences noted as * p≤0.05, ** p≤0.01, or *** p≤0.0001.

To test for the generation of VC2-specific cellular immune responses, a CFSE-membrane labeling assay was utilized to detect cellular proliferation of CD4^+^ and CD8^+^ T cells in the presence of a pool of specific peptides representing known or predicted CD4^+^ and CD8^+^ T epitopes (Table S1). Splenocytes from vaccinated mice produced both CD8^+^ and CD4^+^ T cell proliferation, while lymphocytes from mock-vaccinated mice did not respond to the pooled peptide stimuli (Fig. 8A). Additional analysis was performed by determining the relative levels of Th1/Th2 cytokines in response to the peptide pool. Significant induction of IFNγ, TNFα, IL-4 and IL-5 were noted, while IL-2 was not significantly induced in the vaccinated versus mock-vaccinated mice (Fig. 8:B–F).

## Discussion

Previous studies from our laboratory have focused on the role of HSV-1 gK in neuronal entry and spread. Capitalizing on our findings that gK is necessary for infection of ganglionic neurons after ocular infection, we explored the use of a gK-null virus as a potential vaccine for HSV-1 and HSV-2 genital infection. This initial work showed that gK-null vaccination produced effective strong cellular immune responses and provided significant protection in mice [54]. However, replication of the gK-null virus requires the use of a complementing cell line, negatively impacting the possibility that this virus could be produced for human use. In contrast, the VC2 engineered virus can efficiently replicate in standard cell cultures, while retaining the *in vivo* avirulent characteristics of the gK-null virus. The VC2 virus was highly immunogenic and conferred 100% protection of vaccinated mice against lethal intravaginal challenge with highly virulent HSV-1 and HSV-2 strains.

### Construction of the VC2 virus

We have shown that gK forms a functional protein complex with the UL20 membrane protein that is required for their intracellular transport, cell-surface expression and ability to modulate gB-mediate membrane fusion [61], [64]. Modulation of gB fusogenic properties is mediated via direct protein-protein interactions between the gK and gB amino termini, as well as between the gB carboxyl-terminus and the UL20 amino terminus [57], [60]. We constructed the VC2 virus under the hypothesis that disruption of gK/UL20 interaction with gB will lead to viral attenuation. This hypothesis was supported by the fact that deletion of 37 aa from the amino terminus of gK prevented the virus from infecting ganglionic neurons after ocular infection of mice. Serial deletions of amino acids 4–22, 4–27, and 4–47 from the amino terminus of the UL20 protein have revealed that amino acids 4–27 are dispensable for virus replication in cell culture with the 4–22 deletion producing similar virus titers to that of the HSV-1(F) parental virus (Chouljenko and Kousoulas, unpublished). Therefore, we chose to engineer the 4–22 UL20 deletion into the VC2 virus providing an additional safety feature, since rescue of the VC2 virus by a wild-type genome *in vivo* would require a double recombination-rescue event to take place. Moreover, disruption of the UL20 amino terminus would provide additional dysregulation of gB functions, as evidenced by the fact that the UL20Δ4-22 mutant virus caused virus-induced cell fusion in cell culture (not shown). However, incorporation of the gKΔ31-68 mutation eliminated the UL20Δ4-22-induced cell fusion providing additional support for our long-standing hypothesis that UL20 and gK cooperate to regulate gB’s fusogenic properties primarily through interactions of the amino-termini of gK and gB [57], [60]. As expected the VC2 virus replicated efficiently in Vero cells in comparison to its parental virus HSV-1(F), while it produced on average smaller viral plaques as has been shown previously for the gKΔ31-68 mutation in both the HSV-1(F) and HSV-1(McKrae) genetic backgrounds [55], [57]. Also, the VC2 virus efficiently utilized the nectin-1 and HVEM receptors for virus entry except the PILRα receptor, as we have shown previously for the HSV-1(F) virus [62].

### Vaccination experiments

We have shown that HSV-1(F) BAC does not cause substantial disease symptoms after ocular infection of mice, in contrast to the highly virulent HSV-1(McKrae) strain, although both viruses are readily transmitted to ganglionic neurons and establish latency [55], [65]. Consistent with these previously reported results, safety experiments performed by intranasal infection with 10^6^ PFU of either HSV-1(F) BAC or VC2 viruses revealed no apparent clinical symptoms and absence of detectable viral genomes in trigeminal ganglia. Similarly, infectious doses of up to 10^7^ PFU of either HSV-1(F) BAC or VC2 delivered intramuscularly did not produce any significant clinical disease symptoms and there was no viral DNA detected in either trigeminal or dorsal root ganglia of infected mice. However, it is possible that very low numbers of viral DNA were present in the tested tissues samples, since the limit of qPCR detection was approximately 3 copies/µl. The presence of high numbers of viral DNA in the positive control sample versus the undetected levels of DNA in the vaccinated animals support the hypothesis that very low amounts, if any of challenging virus was able to infect ganglionic neurons and establish latency.

Glycoprotein K has been associated with increased virulence and immunopathogenesis. Specifically, ocular infection of mice previously vaccinated with gK exacerbated corneal immunopathogenesis, while a HSV-1 virus expressing two copies of the gK gene was significantly more virulent than the wild-type virus [66]–[70]. Recently, we showed that the gKΔ31-68 mutation attenuates the highly virulent ocular HSV-1(McKrae) strain and prevent infection of trigeminal ganglionic neurons after ocular infection [55]. The VC2 vaccine strain contains the gKΔ31-68 mutation, which attenuates the virus and preventing efficient infection of ganglionic neurons. Therefore, it is reasonable to assume that VC2 is substantially safer than HSV-1(F) BAC, since it contains double gene deletions in gK and UL20 associated with defects in neuronal entry.

In general, live-attenuated viral vaccines mimic natural infections and induce more robust humoral and cellular immune responses against a broad spectrum of viral proteins. In contrast, subunit vaccines can only carry a limited number of immunogenic epitopes and do not directly induce innate immune responses, as is the case with live virus vaccines. Intramuscular immunization with the VC2 virus produced neutralizing antibody against both HSV-1(McKrae) and HSV-2(G) virus. HSV-1 and HSV-2 share a relatively high degree of protein sequence homology and are known to induce type-common antibodies (cross reactive between HSV-1 and HSV-2) that recognize major antigenic viral determinants, the majority of which constitute sequential and conformational domains of viral glycoproteins [71], [72]. Moreover, pre-existing HSV-1 exposure is known to reduce the severity and duration of HSV-2 infection [29] suggesting the elicitation of type-common immune responses.

Assessment of T cell responses was facilitated by the use of pools of selected and well-characterized peptides representing gB and gD-specific CD4^+^ andCD8^+^ T cell epitopes, as reported previously [54], including few additional viral peptides (Table S1). Proliferation of T-cells cultured with pooled peptides is a direct measure of memory T cells capable of recognizing the epitopes present in the peptide pool only. As such, they are expected to underestimate the overall T-cell response against the HSV proteome that is expressed by the VC2 vaccine. As we reported previously, the most prominent T cell response observed in gK-null vaccinated mice was from gB-specific CD8^+^T cells (specifically peptide 161–176). Interestingly in human patients, this particular T-cell epitope has been identified as immunodominant, recalling the strongest HLA-DR-dependent CD4^+^ T-cell proliferation and IFN-γ production in contrast to other epitopes [73]. The gB (161–176) and gB (499–506) peptide sequences are identical between HSV-1 and HSV-2, therefore, we hypothesize that immune responses directed toward these two epitopes may contribute to the observed cross-protection against both HSV-1 and HSV-2 infections. In addition, most other peptides utilized for the proliferation assays exhibited a high level of amino acid conservation and homology in the HSV-1 and HSV-2 proteomes suggesting that they may also be involved in the induction of cross-protective immunity.

Intramuscular vaccination with the VC2 virus followed by intra vaginal lethal challenge with either HSV-1 (McKrae) or HSV-2 (G) protected 100% of the vaccinated mice, while all mock-vaccinated mice died. Importantly, infectious virus could not be recovered after immunosuppression of vaccinated and challenged mice and the ganglia of these mice did not contain detectable viral DNA. Collectively these results and the observed rapid inhibition of virus replication in infected vaginal tissues in vaccinated versus mock-vaccinated mice, suggests that protection against the challenging viruses was conferred largely by limiting virus replication in infected vaginal tissues. A potential limitation of the virus reactivation after immunosuppression assay is that approximately 30% of latently-infected animals would be expected to reactivate infectious virus production under similar immunosuppression conditions. In addition, although all immunosuppressed mice exhibited clear clinical signs of immunosuppression under these standard treatment procedures, verification of a substantial reduction in blood lymphocytes would have provided additional evidence of their immune status. Lastly, although qPCR utilized in these studies is sensitive to 3 viral DNA copies per sample, explant cultures of ganglionic tissues may recover very low amounts of virus indicating the presence of low copy of viral DNA in ganglionic neurons. Although not done in this study, it would be of interest to test for virus in an extended explant assay to further support our sensitive qPCR findings.

Adaptive immune responses are essential for providing protection against HSV-1 and HSV-2 infections [28], [74], [75]. Recently, it was shown that HSV-2-specific CD8^+^ T cells generated after chemo-attractant therapy given vaginally in mice mediate long-lived protection against HSV-2 challenge [76]. Our observations of the inhibition of viral replication in vaginal tissues within the first 3–4 days post infection suggests that the induction of neutralizing antibodies and the rapid local recruitment of cytotoxic T-cells are sufficient to protect against HSV infection. This theory is supported by the presence of neutralizing IgG antibodies and the development of potent memory T cell pools in the vaccinated mice. Additional studies are needed to assess the level of tissue-specific, intra-vaginal immunity to HSV-1 and HSV-2 infections after a single dose of VC2.

Ideally, a live-attenuated vaccine could be used for both prophylactic and therapeutic purposes. Elicitation of robust tissue-specific T-cell memory responses would confer substantial advantage in limiting replication of reactivated virus, as well as inhibiting secondary infections. It is also likely, that CD8^+^T cells may prevent viral reactivation from latently infected neurons. The VC2 virus could be effectively utilized as a vector for expression of other viral and bacterial pathogens. VC2 expressed foreign antigens may provide a strong adjuvant effect causing the generation of protective adaptive immune responses against mucosally transmitted pathogens such as HIV and Chlamydia trachomatis.

## Materials and Methods

This work was approved by the Louisiana State University School of Veterinary Medicine IACUC protocol 13-011. Mice were mildly anesthetized by inhalation of 2–3% isoflurane prior to vaccination. Mice were euthanized by cervical dislocation.

### Viruses

VC2 recombinant virus construction was performed using double red recombination system in E. coli SW105 cells as described previously [55]. Briefly, specific oligonucleotides designed to delete (aa 31–68) within the ORF encoding the HSV-1 gene UL53 (gK) were used first to generate the respective BAC cloned into *E. coli.* After transfection of the Vero cells the recombinant HSV-1 virus gK Δ31-68 was recovered. Fresh Vero cells were infected with this virus and circular viral DNA was isolated 6 hours post infection (hpi) using the method previously described by Hirt *et al.* [77]. Virus DNA was electroporated into SW 105 cells and a second round of recombination was performed using specific oligonucleotides designed to delete (aa 4–22) within the ORF encoding HSV-1 gene UL20. Recombinant virus VC2 was recovered after transfection of Vero cells. Double deletions within the gK and UL20 genes were confirmed by capillary DNA sequencing. The absence of any other mutations within all HSV-1 structural proteins was confirmed by NGS sequencing using Ion Torrent Personal Genome Machine. Stocks of VC2, HSV-1 (McKrae), and HSV-2 (G), were grown and titrated in Vero cells.

### Next generation genomic DNA sequencing

DNA sequencing of the HSV-1 VC2 mutant virus was performed using the Ion Torrent Personal Genome Machine (PGM) and the 316 sequencing Chip (Life Technologies). Two independent total DNA samples derived from infected Vero cells and from partially purified virions were isolated using the PureLink Genomic DNA mini Kit (Invitrogen). The Ion Xpress Plus Fragment Library Kit (Life Technologies) was used to prepare high-quality fragment libraries from approximately 1 µg of total DNA. Template-positive Ion Sphere Particles (ISPs) containing clonally amplified DNA were produced using the Ion OneTouch 200 Template Kit v2 DL (for 200 base-read libraries) with the Ion OneTouch instrument. The Ion OneTouch ES instrument was used to enrich ISPs intended for the Ion PGM System using the Ion PGM 200 Sequencing Kit.

### VC2 replication and entry characteristics

Replication kinetics assay was performed on confluent monolayers of green African monkey kidney cells (Vero) in 12 well plates. Infections with either HSV-1 (F) or mutant virus VC2 were performed at an MOI of 0.1 and 5. Inoculated plates were placed at 4°C for 1 hour to allow for virion attachment and returned to 37°C for another hour to allow for entry. Plates were then washed with 1× Phosphate Buffered Saline and a final volume of 1 mL of complete DMEM 10% heat inactivated FBS applied to each. Plates were frozen at −80°C until titrated for the following times post infection; 0, 2, 4, 6, 9, 12, 18, 24, and 36 hours. Samples were titrated on confluent monolayers of Vero cells. Infected cell cultures were fixed 48 hpi using formalin-acetic acid- alcohol (FAA) and stained with crystal violet. Plaques were counted using a light microscope and virion titers expressed as PFU/mL were derived for each sample.

Entry assay into CHO cells expressing known HSV-1 entry receptors was conducted as described in Chowdhury *et al.* [62]. Plaque morphology assays were conducted on confluent monolayers of Vero cells in 6-well formats. Virus was serial diluted until single isolated plaques were visible. Immunohistochemistry was performed using polyclonal rabbit anit-HSV-1 primary antibody (Dako, Denmark), polyclonal goat anti-rabbit immunoglobulins HRP conjugated secondary antibody (Dako, Denmark), and visualized using Vector NovaRED Substrate Kit (Vector, Burlingame, CA). Substrate was allowed to develop until sufficient coloration for microscopic imagery.

### Safety and neurovirulence

Neurovirulence assessment was conducted by inoculation of 20 mice with 10^6^ PFU either intranasall or intramuscularly with 10 in each group. Mice were monitored daily for 20 days post inoculation for the manifestation of disease. On day 21, mice were sacrificed and trigeminal ganglia for intranasal inoculations and dorsal root ganglia (for intramuscular inoculations) were collected. Total tissue DNA was extracted using the Qiagen DNeasy Blood and Tissue Kit (Qiagen) and viral genomes were estimated using quantitative PCR.

### Vaccination

All animal studies were carried out after the appropriate approvals were obtained from the Louisiana State University Institutional Animal Care and Use Committee. Six week old female Balb/c mice (LSU DLAM Breeding Colony, Baton Rouge, LA) were used in this study. Each mouse was identified with an ear tag (National Band and Tag Company, KY, USA). Mice were divided into two groups to receive either the vaccine or mock inoculations. Eighty mice, 40 in each group, were mildly anesthetized by inhalation of 2–3% isoflurane and administered a single 100 µL intramuscular injection of either 1×10^7^ PFU of VC2 or equivalent volume of conditioned media. Mice were then observed daily collecting weight and clinical observations.

### Tissue collection and analysis

On day 21 post vaccination 20 mice from each group were anesthetized by inhalation of 2–3% isoflurane and bled via cardiac stick. Maximum volume of blood was collected and mice were euthanized by cervical dislocation. Blood was allowed to clot at 4°C overnight in 5mL falcon tubes (Becton Dickinson, Franklin lakes, NJ) and serum collected into 2 mL Sarstedt Screw Cap Micro Tubes (Sarstedt inc, Newton, NC) and stored at −20°C until use. Spleens were excised from euthanized animals, minced and passed through a 10 µm nylon mesh cell strainer (Fisher Scientific) in Hank’s Balanced Salt Solution. Cell suspensions were then pelleted by centrifugation at 300 × g for 5 minutes and frozen in 5% DMSO Heat Inactivated Fetal Bovine Serum at a concentration of 10^7^ cells/ml. Cells were stored in liquid nitrogen until use.

Dorsal root ganglia were excised as described in Pazyra-Murphy *et al.* [49] Briefly, the peritoneal cavity of the mouse was opened and eviscerated of all organs. Tissues covering the ventral portion of the spine were removed. Dissection was conducted under magnification with a dissecting microscope. Carefully with curved scissors the spinal column was cut medially and scissors inserted into the subarachnoid space to make two lateral cuts along the length of the spine. Spinal chords were removed with attached dorsal root ganglia. DNA was extracted using the Qiagen DNeasy Blood and Tissue kit (Qiagen Sciences, Maryland, USA) as per the manufactures instructions and DNA was precipitated with an equal volume of isopropenol plus 0.3 M sodium acetate, washed with 70% ethanol, and resuspended to a final volume of 50 µL nuclease free water.

### Polychromatic flow cytometry and analysis

Cryopreserved Cells were resuspended at a concentration of 10^6^ cells/mL and labeled with the membrane stain Carboxyfluorescein succinimidyl ester (CFSE). Labeled cells were then cultured at a concentration of 10^5^ cells/well in a 96 well U-bottom plate and incubated at 37°C and 5% CO_2_ for 7 days in the presence of pooled peptides specific to either HSV-1 or HSV-2 at a concentration of 10 µg/mL Cells were then stained with polyclonal anti-mouse CD4 antibody conjugated to PE (BD Biosciences) and polyclonal anti-mouse CD8a antibody conjugated to APC (BD Biosciences). Proliferation of labeled T cell subsets was assessed using an Accuri C6 personal flow cytometer. Unstimulated CFSE-labeled splenocytes were used to establish the CFSE^bright^ population and to define the gating used to quantify CFSE^dim^ (proliferating) T cells.

Supernatants from cultured spleenocytes were stored at −20°C until analysis. Cytokine responses from cultured splenocytes were analyzed using the BD Cytometric Bead Array (CBA) Mouse Th1/Th2 Cytokine Kit (BD Biosciences, San Diego, CA) read using a Bioplex analyzer (Bio-Rad, Hercules, CA) as per the manufacturer’s instructions.

### Challenge

On day 15 post vaccination mice were administered Depo Provera (Upjohn, Kalamazoo, MI) via intra muscular injection as described previously Iyer *et al*. [54] On the day of challenge mice vaginas were swabbed with sterile polystyrine applicator tips dipped in 100 µL DMEM containing 50 mg/L primocin. 10^6^ plaque forming units of highly virulent HSV-1 (McKrae) or HSV-2 (G) were instilled in the vaginal vault and mice were closely monitored daily for clinical manifestation of disease recording daily weight and clinical scores. Mice were scored on a scale of 0–6 (0 = no disease, 1 = ruffled fur and generalized morbidity, 2 = mild genital erythema and edema, 3 = moderate genital inflammation, 4 = genital inflammation with purulent discharge, 5 = hind limb paralysis, 6 = death). On the day an animal succumbed to disease, or on the day of sacrifice pertinent tissues were collected and preserved in 10% neutral buffered formalin (American Mastertech, Lodi, CA).

### Latency reactivation

Reactivation was conducted as described in Cooke *et al* [63]. Briefly, on day 100 post challenge vaccinated mice, which had already survived challenge, received a series of intravenous injections. First 5 mg of cyclophosphamide (Baxter, Deerfield, IL) followed 24 hours later by 0.2 mg of dexamethasone (Butler Schein, Dublin, OH) injected via the same route. Mice were monitored daily for any clinical manifestation of disease. Vaginal swab samples were taken daily for 5 days prior to and post administration of cyclophosphamide and dexamethasone and assayed by plaque assay for the presence of virus, as described under the virus shedding heading. Surviving mice were sacrificed for analysis of excised nervous tissue for the presence of viral DNA.

### Quantitative PCR

Dorsal root ganglia (DRG) were resected from vaccinated mice and unvaccinated controls, challenged with either HSV1 McKrae or HSV2-G. The DRGs were vigorously aspirated and DNA was extracted using the Qiagen DNeasy Blood & Tissue Kit as per the manufacturer’s instructions. The eluted DNA was quantified using a Nanodrop 1000 spectrophotometer. Equal amounts of DNA from each sample were used to perform quantitative real-time PCR analysis on an Applied Biosystems 7900HT Fast Real-Time PCR System. Viral DNA from purified HSV-1 (McKrae) and HSV-2(G) were used as positive controls. The following primer/probe combinations were used to specifically detect HSV-1 (McKrae) or HSV-2(G) (see also Table 1): 1) HSV1gDFP (ACGTACCTGCGGCTCGTGAAGA); 2) HSV1 Probe (Fam – AGCCAAGGGCTCCTGTAAGTACGCCCT – Tamra); 3) HSV1 gD RP (TCACCCCCTGCTGGTAG GCC); 4) HSV2gDFP (CCGCGGGTACGTACCTGCGGCTAG); 5) HSV2 Probe (HEX - GGCCC GCGC/ZEN/CTCCTGCAAGTACGCTCT – IABkFQ); 6) HSV2 gD RP (GCCCTGTTGGTAGGCCTT CGAGGTG). To determine the sensitivity of the qPCR assay, HSV-1 and HSV-2 genomic DNA were quantified and their respective molar concentrations was calculated using the formula:– {µgDNA × (pmol/660) × (10^6^ pg/1µg) × (1/N) = pmol DNA, where N = number of nucleotides}. Ten-fold serial dilutions ranging from 10^5^–10^0.1^ molecules were used as template samples in Taqman PCR reactions, and water was used a no template control. qPCR was performed on the Applied biosystems 7900HT Fast Real-Time PCR System. HSV target DNA was detected at the lowest dilution (2.7×10^−8^ µg of DNA) containing 3 copies per µl. No viral DNA was detected in the no template control sample. The linear range of detection ranged from 3 to 10^6^ viral DNA copies with a mock-vaccinated mice exhibiting more than 10^6^ viral DNA copies per sample.

### Virus shedding

On the day of challenge, before administration of virus, and daily following innoculation, vaginas were swabbed with sterile polystyrene applicator tips dipped in 100 uL DMEM containing 50 mg/L Primocin (InvivoGen, San Diego, CA). Swab samples were stored at −80°C until titration. Titration of swab samples was conducted on confluent monolayers of Vero cells. Samples were resuspended in 900 µl of DMEM +50 mg/L Primocin for an initial dilution of 10^−1^ and diluted in 10 fold increments out to 10^−6^. 250 µL of each dilution was plated in duplicate and incubated at 24°C for 1 hour. Dilutions were then aspirated and wells were covered with 1% DMEM methylcellulose containing 1% FBS and 50 mg/L Primocin. Plates were then incubated at 37°C with 5% CO_2_ for 36–48 hours until visible plaques had formed. Plates were then fixed with FAA and stained with crystal violet. Plaques were counted at dilutions yielding greater than 20 plaques per well.

### Antibody ELISA and serum neutralization

Relative anti-HSV-1 IgG serum concentrations were quantified using commercially available Mouse/Rat HSV-1 IgG ELISA (Calbiotech, Spring Valley, CA). Serum collected on day 21 was used to neutralize 50 µL of stock HSV-1 Mckrae and HSV-2 G containing approximately 5×10^5^ and 3×10^3^ PFU, respectively. Serum was first diluted 1∶10 in complete DMEM containing 10% heat inactivated FBS. Diluted serum was then two fold serial diluted to 1∶160. 50 µL of stock virus was then added to each dilution of serum, 50 µL each, to a total volume of 100 µL. Addition of virus made serum dilutions 1∶20, 1∶40, 1∶80, 1∶160, and 1∶320. Serum virus mixtures were then placed on a rocker at room temperature for 1 hour and frozen at −80°C until titration on Vero cells.

## Supporting Information

Table S1 **Table of epitopes.** Table of peptides used in pools for spleenocyte stimulation assays.
(DOCX)

## References

[1] Bradley H, Markowitz LE, Gibson T, McQuillan GM (2014) Seroprevalence of herpes simplex virus types 1 and 2–United States, 1999–2010. J Infect Dis 209: 325–333. 24136792 10.1093/infdis/jit458

[2] Hofstetter AM, Rosenthal SL, Stanberry LR (2014) Current thinking on genital herpes. Curr Opin Infect Dis 27: 75–83. 24335720 10.1097/QCO.0000000000000029

[3] Tronstein E, Johnston C, Huang ML, Selke S, Magaret A, et al (2011) Genital shedding of herpes simplex virus among symptomatic and asymptomatic persons with HSV-2 infection. JAMA 305: 1441–1449. 21486977 10.1001/jama.2011.420 PMC3144252

[4] Mertz GJ (2008) Asymptomatic shedding of herpes simplex virus 1 and 2: implications for prevention of transmission. J Infect Dis 198: 1098–1100. 18783317 10.1086/591914

[5] Roberts CM, Pfister JR, Spear SJ (2003) Increasing proportion of herpes simplex virus type 1 as a cause of genital herpes infection in college students. Sex Transm Dis 30: 797–800. 14520181 10.1097/01.OLQ.0000092387.58746.C7

[6] Anuradha K, Singh HM, Gopal KV, Rama Rao GR, Ramani TV, et al (2008) Herpes simplex virus 2 infection: a risk factor for HIV infection in heterosexuals. Indian J Dermatol Venereol Leprol 74: 230–233. 18583789 10.4103/0378-6323.41367

[7] Mugo N, Dadabhai SS, Bunnell R, Williamson J, Bennett E, et al (2011) Prevalence of herpes simplex virus type 2 infection, human immunodeficiency virus/herpes simplex virus type 2 coinfection, and associated risk factors in a national, population-based survey in Kenya. Sex Transm Dis 38: 1059–1066. 21992985 10.1097/OLQ.0b013e31822e60b6

[8] Reynolds SJ, Risbud AR, Shepherd ME, Zenilman JM, Brookmeyer RS, et al (2003) Recent herpes simplex virus type 2 infection and the risk of human immunodeficiency virus type 1 acquisition in India. J Infect Dis 187: 1513–1521. 12721931 10.1086/368357

[9] Renzi C, Douglas JM Jr, Foster M, Critchlow CW, Ashley-Morrow R, et al (2003) Herpes simplex virus type 2 infection as a risk factor for human immunodeficiency virus acquisition in men who have sex with men. J Infect Dis 187: 19–25. 12508142 10.1086/345867

[10] Wald A, Link K (2002) Risk of human immunodeficiency virus infection in herpes simplex virus type 2-seropositive persons: a meta-analysis. J Infect Dis 185: 45–52. 11756980 10.1086/338231

[11] Sartori E, Calistri A, Salata C, Del Vecchio C, Palu G, et al (2011) Herpes simplex virus type 2 infection increases human immunodeficiency virus type 1 entry into human primary macrophages. Virol J 8: 166. 21486479 10.1186/1743-422X-8-166 PMC3083365

[12] Brown JM, Wald A, Hubbard A, Rungruengthanakit K, Chipato T, et al (2007) Incident and prevalent herpes simplex virus type 2 infection increases risk of HIV acquisition among women in Uganda and Zimbabwe. AIDS 21: 1515–1523. 17630545 10.1097/QAD.0b013e3282004929

[13] Freeman EE, Weiss HA, Glynn JR, Cross PL, Whitworth JA, et al (2006) Herpes simplex virus 2 infection increases HIV acquisition in men and women: systematic review and meta-analysis of longitudinal studies. AIDS 20: 73–83. 16327322 10.1097/01.aids.0000198081.09337.a7

[14] Freeman EE, White RG, Bakker R, Orroth KK, Weiss HA, et al (2009) Population-level effect of potential HSV2 prophylactic vaccines on HIV incidence in sub-Saharan Africa. Vaccine 27: 940–946. 19071187 10.1016/j.vaccine.2008.11.074 PMC2686080

[15] Johnston C, Koelle DM, Wald A (2014) Current status and prospects for development of an HSV vaccine. Vaccine 32: 1553–1560. 24016811 10.1016/j.vaccine.2013.08.066 PMC4106293

[16] Gottlieb SL, Low N, Newman LM, Bolan G, Kamb M, et al (2014) Toward global prevention of sexually transmitted infections (STIs): The need for STI vaccines. Vaccine 32: 1527–1535. 24581979 10.1016/j.vaccine.2013.07.087 PMC6794147

[17] Blank H, Haines HG (1973) Experimental human reinfection with herpes simplex virus. J Invest Dermatol 61: 223–225. 4355282 10.1111/1523-1747.ep12676442

[18] Koelle DM, Posavad CM, Barnum GR, Johnson ML, Frank JM, et al (1998) Clearance of HSV-2 from recurrent genital lesions correlates with infiltration of HSV-specific cytotoxic T lymphocytes. J Clin Invest 101: 1500–1508. 9525993 10.1172/JCI1758 PMC508728

[19] Milligan GN, Bernstein DI, Bourne N (1998) T lymphocytes are required for protection of the vaginal mucosae and sensory ganglia of immune mice against reinfection with herpes simplex virus type 2. J Immunol 160: 6093–6100. 9637526

[20] Schiffer JT, Corey L (2013) Rapid host immune response and viral dynamics in herpes simplex virus-2 infection. Nat Med 19: 280–290. 23467247 10.1038/nm.3103 PMC3981536

[21] Wakim LM, Jones CM, Gebhardt T, Preston CM, Carbone FR (2008) CD8(+) T-cell attenuation of cutaneous herpes simplex virus infection reduces the average viral copy number of the ensuing latent infection. Immunol Cell Biol 86: 666–675. 18607387 10.1038/icb.2008.47

[22] Zhu J, Koelle DM, Cao J, Vazquez J, Huang ML, et al (2007) Virus-specific CD8+ T cells accumulate near sensory nerve endings in genital skin during subclinical HSV-2 reactivation. J Exp Med 204: 595–603. 17325200 10.1084/jem.20061792 PMC2137910

[23] Dudley KL, Bourne N, Milligan GN (2000) Immune protection against HSV-2 in B-cell-deficient mice. Virology 270: 454–463. 10793004 10.1006/viro.2000.0298

[24] St Leger AJ, Hendricks RL (2011) CD8+ T cells patrol HSV-1-infected trigeminal ganglia and prevent viral reactivation. J Neurovirol 17: 528–534. 22161682 10.1007/s13365-011-0062-1

[25] Li Z, Palaniyandi S, Zeng R, Tuo W, Roopenian DC, et al (2011) Transfer of IgG in the female genital tract by MHC class I-related neonatal Fc receptor (FcRn) confers protective immunity to vaginal infection. Proc Natl Acad Sci U S A 108: 4388–4393. 21368166 10.1073/pnas.1012861108 PMC3060240

[26] Morrison LA, Zhu L, Thebeau LG (2001) Vaccine-induced serum immunoglobin contributes to protection from herpes simplex virus type 2 genital infection in the presence of immune T cells. J Virol 75: 1195–1204. 11152492 10.1128/JVI.75.3.1195-1204.2001 PMC114025

[27] Seppanen M, Meri S, Notkola IL, Seppala IJ, Hiltunen-Back E, et al (2006) Subtly impaired humoral immunity predisposes to frequently recurring genital herpes simplex virus type 2 infection and herpetic neuralgia. J Infect Dis 194: 571–578. 16897653 10.1086/506477

[28] Dropulic LK, Cohen JI (2012) The challenge of developing a herpes simplex virus 2 vaccine. Expert Rev Vaccines 11: 1429–1440. 23252387 10.1586/erv.12.129 PMC3593236

[29] Koelle DM, Corey L (2003) Recent progress in herpes simplex virus immunobiology and vaccine research. Clin Microbiol Rev 16: 96–113. 12525427 10.1128/CMR.16.1.96-113.2003 PMC145296

[30] Roth K, Ferreira VH, Kaushic C (2013) HSV-2 vaccine: current state and insights into development of a vaccine that targets genital mucosal protection. Microb Pathog 58: 45–54. 23159485 10.1016/j.micpath.2012.11.001

[31] Rupp R, Bernstein DI (2008) The potential impact of a prophylactic herpes simplex vaccine. Expert Opin Emerg Drugs 13: 41–52. 18321147 10.1517/14728214.13.1.41

[32] Zhu XP, Muhammad ZS, Wang JG, Lin W, Guo SK, et al (2014) HSV-2 vaccine: current status and insight into factors for developing an efficient vaccine. Viruses 6: 371–390. 24469503 10.3390/v6020371 PMC3939461

[33] Belshe RB, Leone PA, Bernstein DI, Wald A, Levin MJ, et al (2012) Efficacy results of a trial of a herpes simplex vaccine. N Engl J Med 366: 34–43. 22216840 10.1056/NEJMoa1103151 PMC3287348

[34] Belshe RB, Heineman TC, Bernstein DI, Bellamy AR, Ewell M, et al (2014) Correlate of Immune Protection Against HSV-1 Genital Disease in Vaccinated Women. J Infect Dis 209: 828–836. 24285844 10.1093/infdis/jit651 PMC3935479

[35] Awasthi S, Friedman HM (2014) A paradigm shift: vaccine-induced antibodies as an immune correlate of protection against herpes simplex virus type 1 genital herpes. J Infect Dis 209: 813–815. 24285847 10.1093/infdis/jit658

[36] Dolan A, Jamieson FE, Cunningham C, Barnett BC, McGeoch DJ (1998) The genome sequence of herpes simplex virus type 2. J Virol 72: 2010–2021. 9499055 10.1128/jvi.72.3.2010-2021.1998 PMC109494

[37] Brittle EE, Wang F, Lubinski JM, Bunte RM, Friedman HM (2008) A replication-competent, neuronal spread-defective, live attenuated herpes simplex virus type 1 vaccine. J Virol 82: 8431–8441. 18562543 10.1128/JVI.00551-08 PMC2519657

[38] Awasthi S, Zumbrun EE, Si H, Wang F, Shaw CE, et al (2012) Live attenuated herpes simplex virus 2 glycoprotein E deletion mutant as a vaccine candidate defective in neuronal spread. J Virol 86: 4586–4598. 22318147 10.1128/JVI.07203-11 PMC3318599

[39] Prichard MN, Kaiwar R, Jackman WT, Quenelle DC, Collins DJ, et al (2005) Evaluation of AD472, a live attenuated recombinant herpes simplex virus type 2 vaccine in guinea pigs. Vaccine 23: 5424–5431. 15950327 10.1016/j.vaccine.2005.02.028 PMC2718572

[40] van Lint AL, Torres-Lopez E, Knipe DM (2007) Immunization with a replication-defective herpes simplex virus 2 mutant reduces herpes simplex virus 1 infection and prevents ocular disease. Virology 368: 227–231. 17915278 10.1016/j.virol.2007.08.030 PMC2099303

[41] Dudek T, Mathews LC, Knipe DM (2008) Disruption of the U(L)41 gene in the herpes simplex virus 2 dl5-29 mutant increases its immunogenicity and protective capacity in a murine model of genital herpes. Virology 372: 165–175. 18006033 10.1016/j.virol.2007.10.014 PMC2323115

[42] Hoshino Y, Pesnicak L, Dowdell KC, Lacayo J, Dudek T, et al (2008) Comparison of immunogenicity and protective efficacy of genital herpes vaccine candidates herpes simplex virus 2 dl5-29 and dl5-29-41L in mice and guinea pigs. Vaccine 26: 4034–4040. 18565628 10.1016/j.vaccine.2008.05.022 PMC2564964

[43] Da Costa XJ, Morrison LA, Knipe DM (2001) Comparison of different forms of herpes simplex replication-defective mutant viruses as vaccines in a mouse model of HSV-2 genital infection. Virology 288: 256–263. 11601897 10.1006/viro.2001.1094

[44] Halford WP, Puschel R, Gershburg E, Wilber A, Gershburg S, et al (2011) A live-attenuated HSV-2 ICP0 virus elicits 10 to 100 times greater protection against genital herpes than a glycoprotein D subunit vaccine. PLoS One 6: e17748. 21412438 10.1371/journal.pone.0017748 PMC3055896

[45] Akhrameyeva NV, Zhang P, Sugiyama N, Behar SM, Yao F (2011) Development of a glycoprotein D-expressing dominant-negative and replication-defective herpes simplex virus 2 (HSV-2) recombinant viral vaccine against HSV-2 infection in mice. J Virol 85: 5036–5047. 21389121 10.1128/JVI.02548-10 PMC3126160

[46] Brans R, Akhrameyeva NV, Yao F (2009) Prevention of genital herpes simplex virus type 1 and 2 disease in mice immunized with a gD-expressing dominant-negative recombinant HSV-1. J Invest Dermatol 129: 2470–2479. 19357711 10.1038/jid.2009.86 PMC2786208

[47] Brans R, Yao F (2010) Immunization with a dominant-negative recombinant Herpes Simplex Virus (HSV) type 1 protects against HSV-2 genital disease in guinea pigs. BMC Microbiol 10: 163. 20525279 10.1186/1471-2180-10-163 PMC2889954

[48] Augustinova H, Hoeller D, Yao F (2004) The dominant-negative herpes simplex virus type 1 (HSV-1) recombinant CJ83193 can serve as an effective vaccine against wild-type HSV-1 infection in mice. J Virol 78: 5756–5765. 15140973 10.1128/JVI.78.11.5756-5765.2004 PMC415800

[49] Murphy CG, Lucas WT, Means RE, Czajak S, Hale CL, et al (2000) Vaccine protection against simian immunodeficiency virus by recombinant strains of herpes simplex virus. J Virol 74: 7745–7754. 10933680 10.1128/jvi.74.17.7745-7754.2000 PMC112303

[50] Watanabe D, Brockman MA, Ndung'u T, Mathews L, Lucas WT, et al (2007) Properties of a herpes simplex virus multiple immediate-early gene-deleted recombinant as a vaccine vector. Virology 357: 186–198. 16996101 10.1016/j.virol.2006.08.015

[51] Harrington KJ, Hingorani M, Tanay MA, Hickey J, Bhide SA, et al (2010) Phase I/II study of oncolytic HSV GM-CSF in combination with radiotherapy and cisplatin in untreated stage III/IV squamous cell cancer of the head and neck. Clin Cancer Res 16: 4005–4015. 20670951 10.1158/1078-0432.CCR-10-0196

[52] David AT, Baghian A, Foster TP, Chouljenko VN, Kousoulas KG (2008) The herpes simplex virus type 1 (HSV-1) glycoprotein K(gK) is essential for viral corneal spread and neuroinvasiveness. Curr Eye Res 33: 455–467. 18568883 10.1080/02713680802130362

[53] David AT, Saied A, Charles A, Subramanian R, Chouljenko VN, et al (2012) A herpes simplex virus 1 (McKrae) mutant lacking the glycoprotein K gene is unable to infect via neuronal axons and egress from neuronal cell bodies. MBio 3: e00144–00112. 22829677 10.1128/mBio.00144-12 PMC3413403

[54] Iyer AV, Pahar B, Chouljenko VN, Walker JD, Stanfield B, et al (2013) Single dose of Glycoprotein K (gK)-deleted HSV-1 live-attenuated virus protects mice against lethal vaginal challenge with HSV-1 and HSV-2 and induces lasting T cell memory immune responses. Virol J 10: 317. 24165088 10.1186/1743-422X-10-317 PMC3826548

[55] Saied AA, Chouljenko VN, Subramanian R, Kousoulas KG (2014) A replication competent HSV-1(McKrae) with a mutation in the amino-terminus of glycoprotein K (gK) is unable to infect mouse trigeminal ganglia after cornea infection. Curr Eye Res 39: 596–603. 24401006 10.3109/02713683.2013.855238

[56] Tanaka M, Kagawa H, Yamanashi Y, Sata T, Kawaguchi Y (2003) Construction of an excisable bacterial artificial chromosome containing a full-length infectious clone of herpes simplex virus type 1: viruses reconstituted from the clone exhibit wild-type properties in vitro and in vivo. J Virol 77: 1382–1391. 12502854 10.1128/JVI.77.2.1382-1391.2003 PMC140785

[57] Chouljenko VN, Iyer AV, Chowdhury S, Chouljenko DV, Kousoulas KG (2009) The amino terminus of herpes simplex virus type 1 glycoprotein K (gK) modulates gB-mediated virus-induced cell fusion and virion egress. J Virol 83: 12301–12313. 19793812 10.1128/JVI.01329-09 PMC2786757

[58] Lee HC, Chouljenko VN, Chouljenko DV, Boudreaux MJ, Kousoulas KG (2009) The herpes simplex virus type 1 glycoprotein D (gD) cytoplasmic terminus and full-length gE are not essential and do not function in a redundant manner for cytoplasmic virion envelopment and egress. J Virol 83: 6115–6124. 19357164 10.1128/JVI.00128-09 PMC2687392

[59] Kim IJ, Chouljenko VN, Walker JD, Kousoulas KG (2013) Herpes simplex virus 1 glycoprotein M and the membrane-associated protein UL11 are required for virus-induced cell fusion and efficient virus entry. J Virol 87: 8029–8037. 23678175 10.1128/JVI.01181-13 PMC3700202

[60] Chouljenko VN, Iyer AV, Chowdhury S, Kim J, Kousoulas KG (2010) The herpes simplex virus type 1 UL20 protein and the amino terminus of glycoprotein K (gK) physically interact with gB. J Virol 84: 8596–8606. 20573833 10.1128/JVI.00298-10 PMC2919038

[61] Foster TP, Chouljenko VN, Kousoulas KG (2008) Functional and physical interactions of the herpes simplex virus type 1 UL20 membrane protein with glycoprotein K. J Virol. 82: 6310–6323. 10.1128/JVI.00147-08 PMC2447060 18434401

[62] Chowdhury S, Chouljenko VN, Naderi M, Kousoulas KG (2013) The amino terminus of herpes simplex virus 1 glycoprotein K is required for virion entry via the paired immunoglobulin-like type-2 receptor alpha. J Virol 87: 3305–3313. 23302878 10.1128/JVI.02982-12 PMC3592154

[63] Cook SD, Paveloff MJ, Doucet JJ, Cottingham AJ, Sedarati F, et al (1991) Ocular herpes simplex virus reactivation in mice latently infected with latency-associated transcript mutants. Invest Ophthalmol Vis Sci 32: 1558–1561. 1849874

[64] Foster TP, Melancon JM, Olivier TL, Kousoulas KG (2004) Herpes simplex virus type 1 glycoprotein K and the UL20 protein are interdependent for intracellular trafficking and trans-Golgi network localization. J Virol 78: 13262–13277. 15542677 10.1128/JVI.78.23.13262-13277.2004 PMC525009

[65] Kim IJ, Saied AA, Chouljenko VN, Subramanian R, Kousoulas KG (2014) Functional Hierarchy of Herpes Simplex Virus Type-1 Membrane Proteins in Corneal Infection and Virus Transmission to Ganglionic Neurons. Curr Eye Res (In Press). 10.3109/02713683.2014.906626 24749493

[66] Allen SJ, Mott KR, Ghiasi H (2014) Overexpression of herpes simplex virus glycoprotein K (gK) alters expression of HSV receptors in ocularly-infected mice. Invest Ophthalmol Vis Sci 55: 2442–2451. 24667863 10.1167/iovs.14-14013 PMC3989088

[67] Allen SJ, Mott KR, Ljubimov AV, Ghiasi H (2010) Exacerbation of corneal scarring in HSV-1 gK-immunized mice correlates with elevation of CD8+CD25+ T cells in corneas of ocularly infected mice. Virology 399: 11–22. 20079918 10.1016/j.virol.2009.12.011 PMC2830294

[68] Ghiasi H, Perng GC, Nesburn AB, Wechsler SL (2000) Antibody-dependent enhancement of HSV-1 infection by anti-gK sera. Virus Res 68: 137–144. 10958985 10.1016/s0168-1702(00)00165-9

[69] Mott KR, Chentoufi AA, Carpenter D, BenMohamed L, Wechsler SL, et al (2009) The role of a glycoprotein K (gK) CD8+ T-cell epitope of herpes simplex virus on virus replication and pathogenicity. Invest Ophthalmol Vis Sci 50: 2903–2912. 19168902 10.1167/iovs.08-2957

[70] Mott KR, Perng GC, Osorio Y, Kousoulas KG, Ghiasi H (2007) A recombinant herpes simplex virus type 1 expressing two additional copies of gK is more pathogenic than wild-type virus in two different strains of mice. J Virol 81: 12962–12972. 17898051 10.1128/JVI.01442-07 PMC2169076

[71] Kousoulas KG, Huo B, Pereira L (1988) Antibody-resistant mutations in cross-reactive and type-specific epitopes of herpes simplex virus 1 glycoprotein B map in separate domains. Virology 166: 423–431. 2459843 10.1016/0042-6822(88)90513-2

[72] Pereira L, Klassen T, Baringer JR (1980) Type-common and type-specific monoclonal antibody to herpes simplex virus type 1. Infect Immun 29: 724–732. 6260657 10.1128/iai.29.2.724-732.1980 PMC551186

[73] Chentoufi AA, Binder NR, Berka N, Durand G, Nguyen A, et al (2008) Asymptomatic human CD4+ cytotoxic T-cell epitopes identified from herpes simplex virus glycoprotein B. J Virol. 82: 11792–11802. 10.1128/JVI.00692-08 PMC2583686 18799581

[74] Coleman JL, Shukla D (2013) Recent advances in vaccine development for herpes simplex virus types I and II. Hum Vaccin Immunother 9: 729–735. 23442925 10.4161/hv.23289 PMC3903888

[75] Dervillez X, Gottimukkala C, Kabbara KW, Nguyen C, Badakhshan T, et al (2012) Future of an “Asymptomatic” T-cell Epitope-Based Therapeutic Herpes Simplex Vaccine. Future Virol 7: 371–378. 22701511 10.2217/fvl.12.22 PMC3372919

[76] Shin H, Iwasaki A (2012) A vaccine strategy that protects against genital herpes by establishing local memory T cells. Nature 491: 463–467. 23075848 10.1038/nature11522 PMC3499630

[77] Hirt B (1967) Selective extraction of polyoma DNA from infected mouse cell cultures. J Mol Biol 26: 365–369. 4291934 10.1016/0022-2836(67)90307-5

