# Supplementary material for: A Single Intramuscular Vaccination of Mice with the HSV-1 VC2 Virus with Mutations in the Glycoprotein K and the Membrane Protein UL20 Confers Full Protection against Lethal Intravaginal Challenge with Virulent HSV-1 and HSV-2 Strains
Source: PLoS One. 2014 Oct 28;9(10):e109890. doi: 10.1371/journal.pone.0109890 (PMC4211657; doi:10.1371/journal.pone.0109890)
Supplement: Table S1 — Table of epitopes. Table of peptides used in pools for spleenocyte stimulation assays. (DOCX) [file pone.0109890.s001.docx]

| **Virus** | **Glycoprotein** | **Locus** | **Amino Acids** | **References** |
| --- | --- | --- | --- | --- |
| HSV-1 | gD | 70-78 | SLPITVYYA | Chentoufi AA, J Immunol 2010; 184:2561-71 |
| HSV-2 | gD | 77-85 | SIPITVYYA | NA |
| HSV-1 | gD | 270-287 | YTSTLLPPELSETPN | NA |
| HSV-2 | gD | 270-287 | YTSTLLPPELSDTTN | Cooper D, Cell Immunol 2006; 239:113-20 |
| HSV-1 | gD | 278-286 | ALLEDPVGT | Chentoufi AA, J Immunol 2010; 184:2561-71 |
| HSV-2 | gD | 250-258 | ALLEDPAGT | NA |
| HSV-1 | gB | 566-580 | HVNDMLGRVAIAWCE | NA |
| HSV-2 | gB | 562-576 | HVNDMLGRIAVAWCE | Posavad CM, Vaccine 2011; 29:7058-66 |
| HSV-1/HSV-2 | gB | 161-176 | ATMYYKDVTVSQVWF | Chentoufi AA, J Immunol 2010; 184:2561-71 |
| HSV-1/HSV-2 | gB | 499-506 | SSIEFARL | Leger A, J Immunol 2011; 186:3927-33 |
